# Simple, Fast and Selective Detection of Adenosine Triphosphate at Physiological pH Using Unmodified Gold Nanoparticles as Colorimetric Probes and Metal Ions as Cross-Linkers

**DOI:** 10.3390/s121115078

**Published:** 2012-11-06

**Authors:** Dehua Deng, Ning Xia, Sujuan Li, Chunying Xu, Ting Sun, Huan Pang, Lin Liu

**Affiliations:** 1 College of Chemistry and Chemical Engineering, Anyang Normal University, Anyang 455000, Henan, China; E-Mails: ddh@aynu.edu.cn (D.D.); lisujuan1981@gmail.com (S.L.); chunyingxu@126.com (C.X.); sunt725726@163.com (T.S.); 2 College of Chemistry and Chemical Engineering, Central South University, Changsha 410083, Hunan, China

**Keywords:** gold nanoparticles, adenosine triphosphate, colorimetric assay, metal ions

## Abstract

We report a simple, fast and selective colorimetric assay of adenosine triphosphate (ATP) using unmodified gold nanoparticles (AuNPs) as probes and metal ions as cross-linkers. ATP can be assembled onto the surface of AuNPs through interaction between the electron-rich nitrogen atoms and the electron-deficient surface of AuNPs. Accordingly, Cu^2+^ ions induce a change in the color and UV/Vis absorbance of AuNPs by coordinating to the triphosphate groups and a ring nitrogen of ATP. A detection limit of 50 nM was achieved, which is comparable to or lower than that achievable by the currently used electrochemical, spectroscopic or chromatographic methods. The theoretical simplicity and high selectivity reported herein demonstrated that AuNPs-based colorimetric assay could be applied in a wide variety of fields by rationally designing the surface chemistry of AuNPs. In addition, our results indicate that ATP-modified AuNPs are less stable in Cu^2+^, Cd^2+^ or Zn^2+^-containing solutions due to the formation of the corresponding dimeric metal-ATP complexes.

## 1. Introduction

Adenosine triphosphate (ATP) is known to be the universal energy currency in all biological systems and to contribute to cell metabolism, biochemical reactions and active transport. Measurement of cellular ATP level is crucial as this allows us to assess its metabolic state and the degree of contamination of food and medical instruments [1]. For the accurate detection of ATP, the currently used methods, such as high performance liquid chromatography (HPLC) [2], chemiluminescence [3], fluorescence [4], electrochemistry [5], mass spectrometry [6], and enzymatic assays [7], are usually time-consuming, lacking sensitivity or needing complicated instruments. In addition, ATP is an unstable molecule that rapidly hydrolyses to adenosine diphosphate (ADP) and phosphate. Thus, development of a simple, fast and selective ATP detection method remains a great challenge. Recently, molecular-recognition and sensing systems for biological species analysis has attracted much attention from researchers and some aptamer methods have been established for the detection of ATP [8–20]. Unfortunately, these sensors display low selectivity and/or sensitivity or require relatively complicated processes for ATP detection.

Due to the unique size-dependent optical properties of gold nanoparticles (AuNPs), AuNP-based colorimetric assays have been widely applied in a variety of research fields, such as the screening of kinase activity and the measurement of the concentrations of nucleic acid, proteins, metal ions and other small molecules [21–30]. Such methods are very promising in that they involve very simple sample handling procedures and minimum instrumental investments and can be conducted in the field with portable devices. Herein, we report the simple, fast and selective detection of ATP based on the aggregation and color change of AuNPs. This method is based on the following facts: (1) the adenine group of ATP can absorb onto the surface of AuNPs by coordinating interactions between the electron-rich nitrogen atoms and the electron-deficient surface of AuNPs [31–34], and (2) ATP is prone to form dimeric [M_2_(ATP)]_2_ complexes, where M represents a bivalent metal ion (e.g., Cd^2+^, Cu^2+^ and Zn^2+^) [35–39]. Aggregation and color change of the AuNPs were anticipated to occur when ATP and Cu^2+^ were supplemented successively to a solution of AuNPs. The present method is selective to ATP and is much faster and simpler than the existing methods, without the need for expensive and complicated instruments.

## 2. Experimental Section

### 2.1. Apparatus

The photographs were taken with a Sony Cyber-shot digital camera. The UV/Vis spectra were recorded using a Cary 50 spectrophotometer with a 1 cm quartz spectrophotometer cell. The morphology of AuNPs was observed by a FEI Tecnai G2 T20 transmission electron microscope (TEM). Deionized water was purified by a Millipore system (Simplicity Plus, Millipore Corp.).

### 2.2. Reagents

ATP, adenosine diphosphate (ADP), adenosine monophosphate (AMP), guanosine triphosphate (GTP), cytidine triphosphate (CTP), NTP mixture and boric acid were purchased from Sangon Biotech Co., Ltd. (Shanghai, China). Other reagents were obtained from Beijing Chemicals, Ltd. (Beijing, China). All of the chemicals were analytical-grade reagents and were used without further purification. Phosphate (NaH_2_PO_4_ and Na_2_HPO_4_) was used to adjust the pH of boric acid to the desired values, as reported previously [40,41]. NTP mixture was diluted 1,000 fold with borate buffer before assay.

### 2.3. Synthesis of AuNPs

All glassware used in the following procedures was cleaned in a bath of freshly prepared 1:3 HNO_3_–HCl, rinsed thoroughly with water and dried in air prior to use. The citrated-stabilized AuNPs were prepared using a trisodium citrate reduction method as reported previously [42]. Briefly, trisodium citrate (5 mL, 38.8 mM) was rapidly added to a boiling solution of HAuCl_4_ (50 mL, 1 mM), and the solution was boiled continually for an additional 30 min to yield a wine-red solution. After filtering the solution through a 0.45-μm membrane filter to remove the precipitate, the filtrate was stored in a refrigerator at 4 °C for use.

### 2.4. Detection of ATP

A 500 μL dispersion of AuNPs was added to 1 mL 5 mM buffer solution (pH 7.0). ATP at the desired concentration was introduced into the AuNP solution. The incubation time for AuNPs and ATP was 15 min at shake. Then, 30 μM Cu^2+^ was added to the ATP-containing AuNPs solution. Color change and absorption spectra were observed with the naked eye and recorded with UV/Vis spectrometer, respectively. Reaction and detection were conducted at room temperature.

## 3. Results and Discussion

### 3.1. Mechanism of ATP Detection

**Figure 1.**
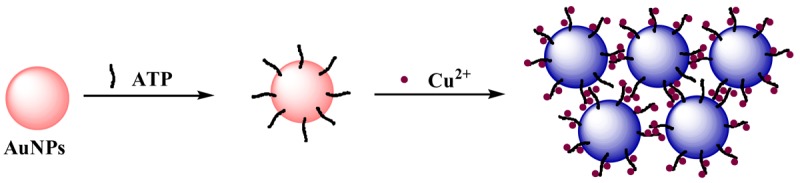
Schematic illustration of the strategy of ATP detection using AuNPs as indicators and Cu^2+^ ions as cross-linkers.

Recently, unmodified AuNPs have been applied to detect DNA, metal ions and other small molecules as a simple, fast and label-free colorimetric method [26,43–47]. The ring nitrogen of hybrid aromatics and primary amines with electron-rich nitrogen atoms are more likely to be bound onto the surface of metal nanoparticles through the coordinating interactions with the electron-deficient surface of metal nanoparticles [43]. ATP with multiple binding sites, including one exocyclic amino group and/or two double-nitrogen hybrid rings, can strongly coordinate to AuNPs by ligand exchange with weakly surface-bound citrate ions [32]. Metal ions, especially Cu^2+^, Cd^2+^ and Zn^2+^, can form dimeric metal-ATP complexes by coordinating to the triphosphate groups and an adenine ring nitrogen [39]. The stability of AuNPs will decrease drastically after the addition of ATP and Cu^2+^, resulting in the occurrence of AuNP aggregation (Figure 1). The molecular linker-based aggregation offers a possible approach to a simple and fast colorimetric assay for the detection of ATP, which does not require specific acceptors.

### 3.2. Color Assay for ATP

**Figure 2.**
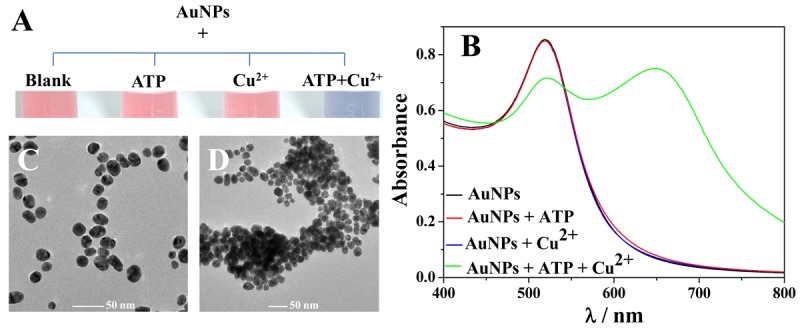
Visual color change (**A**), UV/Vis absorption spectra (**B**) and TEM images (**C**,**D**) of AuNPs in the absence and presence of ATP and/or Cu^2+^. The concentrations of ATP and Cu^2+^ are 15 and 30 μM respectively.

As shown in Figure 2, the AuNPs were red in color and exhibited an absorption peak at 520 nm (A_520_), which was ascribed to its surface plasmon resonance (vial 1 and black curve). No obvious change was observed upon the addition of ATP (vial 2) or Cu^2+^ (vial 3) alone. However, when ATP and Cu^2+^ successively were added to an aqueous suspension of AuNPs, an obvious color change from red to blue was observed (vial 4). Meanwhile, the ATP-induced aggregation of AuNPs was also monitored by UV/Vis spectroscopy (Figure 2(B)). With the addition of Cu^2+^ and ATP, the original absorbance of AuNPs at 520 nm decreased while a new absorbance at ~650 nm (A_650_) increased obviously. These results were further confirmed by the TEM observations: the monodisperse AuNPs in the absence of ATP and Cu^2+^ (Figure 2(C)) and the significant aggregation of AuNPs in the presence of ATP and Cu^2+^ (Figure 2(D)).

### 3.3. Effect of pH

**Figure 3.**
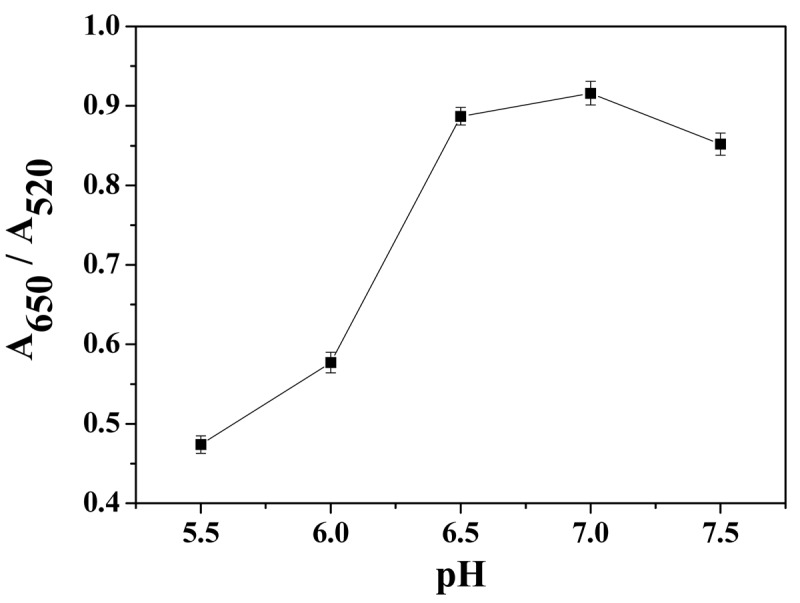
Effect of pH on the A_650_/A_520_ ratio.

Solution pH affects not only the stability of AuNPs and ATP as well as the binding of ATP to AuNPs, but also the formation of the dimeric metal-ATP complex [39]. Therefore, the effect of pH on the A_650_/A_520_ ratio was examined over a range from 5.5 to 7.5. As shown in Figure 3, A_650_/A_520_ reaches a maximum at pH 7.0. Thus, we chose pH 7.0 borate buffer solution as the reaction medium. The signal decreases remarkably at pH values below 6.5, probably due to the lesser stability of ATP and poor binding of ATP to AuNPs and /or Cu^2+^ at low pH.

### 3.4. Sensitivity of AuNPs Suspension to ATP

**Figure 4.**
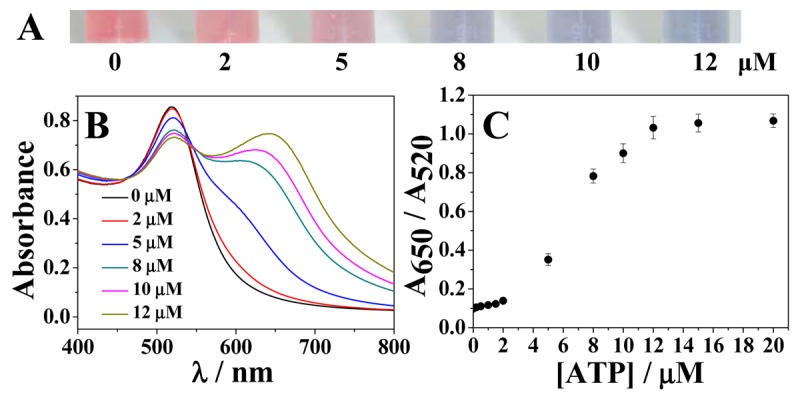
(**A**) Color change with the increase of ATP concentrations. (**B**) Absorbance response for different concentrations of ATP. (**C**) The corresponding plot of A_650_/A_520_ *versus* ATP concentration from 0, 0.1, 0.2, 0.5, 1.0, 1.5, 2, 5, 8, 10, 12, 15 to 20 μM.

To demonstrate the performance of the sensor for naked eye detection of ATP by the mechanism mentioned above, different amounts of ATP were added to the solution of AuNPs followed by addition of Cu^2+^ and the results are presented in Figure 4(A). Upon the addition of increasing concentrations of ATP, the color of AuNPs gradually changed initially from wine red, then to purple, and finally to blue. These results were further conformed by UV/Vis spectroscopy. As shown in Figure 4(B), with the addition of an increasing concentration of ATP to the solution of AuNPs, an obvious decrease in the absorption peak at 520 nm and a strong increase in the absorption peak at 650 nm were clearly detected. The A_650_/A_520_ ratio increased with the increase of ATP concentration (Figure 4(C)). A linear relationship was found between the A_650_/A_520_ ratio and concentration of ATP over the range of 0.1–12 μM: the A_650_/A_520_ ratio slightly increased in the ATP concentration range of 0.1–2 μM and a much more significant intensity increase was observed in the concentration range of 2–12 μM. The detection limit for ATP was determined to be approximately 50 nM (n = 11). This value is comparable to (or even lower than) conventional analytical methods, such as HPLC, chemiluminescence, fluorescence, electrochemistry and mass spectrometry [2–6].

### 3.5. Sensitivity of AuNPs Suspension to ATP

**Figure 5.**
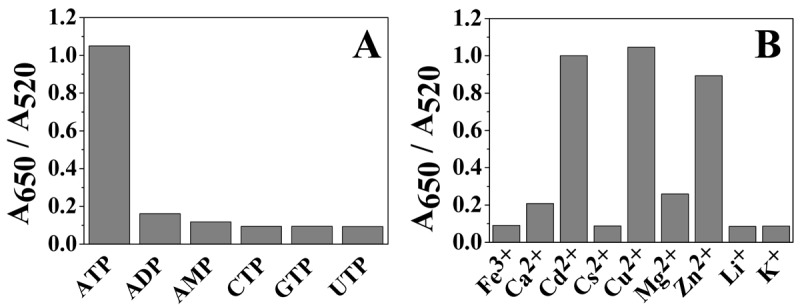
The A_650_/A_520_ ratio of AuNPs in different systems: (**A**) ATP/interferences + Cu^2+^ and (**B**) ATP + different metal ions. The concentrations of ATP, ATP analogs and metal ions are 15, 15 and 30 μM, respectively.

Furthermore, the selectivity of the present approach was evaluated by monitoring the absorbance of the AuNPs in the presence of other biomolecules with similar structures. Our results showed excellent selectivity for ATP over ADP, AMP, GTP, and CTP (Figure 5(A)). We also found that phosphate derivatives such as pyrophosphate phosphate did not induce the aggregation and color change of AuNPs at a concentration of 15 μM. This excellent selectivity was mainly attributable to the metal-binding property of triphosphate groups of ATP with Cu^2+^ [39] and the high adsorption of adenine onto the surface of the AuNPs [33,34]. Moreover, the effect of other metal ions that can bind to ATP on the aggregation of ATP-capped AuNPs was also investigated and the results are shown in Figure 5(B).

### 3.6. Detection of ATP in NTP Mixture

To demonstrate the viability of the assay to measure real ATP samples, we carried out the measurement of amounts of ATP in commercial NTP mixtures. The mixture was treated as in the aforementioned procedures. The level of ATP in the sample was found to be 9.7 ± 0.5 mM, which is close to the expected content of 10 mM.

## 4. Conclusions/Outlook

In conclusion, we reported that ATP can be detected based on the aggregation and color change of bare AuNPs with the aid of metal ions as molecular cross-linkers. A detection limit of 50 nM was achieved, which is comparable to or lower than that achievable by currently used electrochemical, spectroscopic or chromatographic methods. The theoretical simplicity and high selectivity reported herein demonstrate that our AuNPs-based colorimetric assay could be applied in a wide variety of fields by rationally designing the surface chemistry of AuNPs. In addition, our results indicate that ATP-modified AuNPs are less stable in Cu^2+^, Cd^2+^ or Zn^2+^-containing solutions due to the formation of the dimeric metal-ATP complexes.
